# African origin for Madagascan dogs revealed by mtDNA analysis

**DOI:** 10.1098/rsos.140552

**Published:** 2015-05-20

**Authors:** Arman Ardalan, Mattias C. R. Oskarsson, Barbara van Asch, Elisabeth Rabakonandriania, Peter Savolainen

**Affiliations:** 1 Department of Gene Technology, KTH–Royal Institute of Technology, Science for Life Laboratory, Solna 171 21, Sweden; 2 Division of Animal Biotechnology and Genomics, National Institute of Genetic Engineering and Biotechnology (NIGEB), Tehran 14965/161, Iran; 3 IPATIMUP, Rua Dr Roberto Frias s/n, Porto 4200–465, Portugal; 4 Department of Genetics, Faculty of AgriSciences, Stellenbosch University, Stellenbosch 7601, South Africa; 5 Département de Biologie et Écologie Végétales, Faculté des Sciences Université d'Antananarivo, Antananarivo 101, Madagascar

**Keywords:** *Canis familiaris*, mtDNA, Madagascar, Austronesian expansion, Indian Ocean exchange, cultural diffusion

## Abstract

Madagascar was one of the last major land masses to be inhabited by humans. It was initially colonized by Austronesian speaking Indonesians 1500–2000 years ago, but subsequent migration from Africa has resulted in approximately equal genetic contributions from Indonesia and Africa, and the material culture has mainly African influences. The dog, along with the pig and the chicken, was part of the Austronesian Neolithic culture, and was furthermore the only domestic animal to accompany humans to every continent in ancient times. To illuminate Madagascan cultural origins and track the initial worldwide dispersal of dogs, we here investigated the ancestry of Madagascan dogs. We analysed mtDNA control region sequences in dogs from Madagascar (*n*=145) and compared it with that from potential ancestral populations in Island Southeast Asia (*n*=219) and sub-Saharan Africa (*n*=493). We found that 90% of the Madagascan dogs carried a haplotype that was also present in sub-Saharan Africa and that the remaining lineages could all be attributed to a likely origin in Africa. By contrast, only 26% of Madagascan dogs shared haplotypes with Indonesian dogs, and one haplotype typical for Austronesian dogs, carried by more than 40% of Indonesian and Polynesian dogs, was absent among the Madagascan dogs. Thus, in contrast to the human population, Madagascan dogs seem to trace their origin entirely from Africa. These results suggest that dogs were not brought to Madagascar by the initial Austronesian speaking colonizers on their transoceanic voyage, but were introduced at a later stage, together with human migration and cultural influence from Africa.

## 2. Introduction

**Figure 1.**
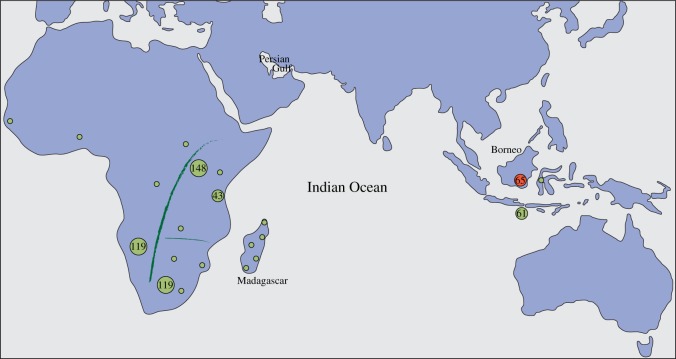
Map showing major sampling regions in Madagascar, Africa and Indonesia. Light green circles represent sampling regions. The number of samples is given for the major sampling regions; minor regions with 6–33 samples are represented by small circles. The sample from Southeast Borneo, the region suggested as the origin for the Austronesian language spoken on Madagascar, is indicated by a red circle. The regions designated as West, upper East and lower East sub-Saharan Africa are separated by green lines.

The island of Madagascar is situated less than 500 km off the east coast of Africa (figure 1) and was one of the last large landmasses to be populated, 1500–2000 years before present (YBP) [1]. Despite its proximity to Africa, linguistic and genetic evidence indicate that Madagascar was initially colonized by Austronesian speaking peoples from Indonesia, but subsequent migration from Africa has resulted in approximately equal genetic contributions from both regions [2]. The Madagascan culture is mainly influenced by Africa and, except for the language, few clear contributions remain from the initial Indonesian culture [3]. The domestic dog (together with the pig and the chicken) was an important part of the Austronesian culture [4], suggesting that the dog may be one of few remaining contributions of Austronesian culture in today's Madagascar. However, the origin of Madagascan dogs (as well as other domestic animals) remains largely unstudied, and an ancestry from the Austronesian expansion or from subsequent contacts with Africa, or both, is possible. A study of the ancestry of the dogs in Madagascar is, therefore, of importance for illuminating origins of Madagascan culture, as well as for describing the worldwide dispersal of dogs.

Madagascar is a linguistic outlier with an Austronesian speaking population in the otherwise largely Bantu-speaking sub-Saharan East Africa. Starting from Taiwan approximately 5000 YBP and aided by improvements in boat technology, Austronesian languages spread across more than half the globe, to Madagascar in the west and Easter Island in the east, in one of the most extensive geographical expansions of a human population in history [4]. Linguistic evidence suggests that the migration to Madagascar took place in early seventh century AD [2,5,6]. The modern Malagasy language shares most of its basic vocabulary (approx. 90%) with the languages spoken in the Barito River region in southeastern Borneo, thus distinctly pointing out the probable geographical origin of the first colonizers of Madagascar [5–7]. The remaining vocabulary consists of contributions from, e.g. Bantu, Malay and Sanskrit languages [5]. The words for most domesticates, including the dog, have Bantu origin, indicating that the ancestors of the modern Malagasy adopted new words for these animals, even though they were already familiar to them [2,8,9].

Palaeoecological and archaeological evidence (e.g. human-modified bones from extinct animals) indicate human presence on Madagascar more than 2000 YBP [10,11]. However, the earliest direct evidence for human occupation in the form of charcoal fragments in northern Madagascar was dated to 1300–1680 YBP [1], in good agreement with the linguistic evidence. This was shortly preceded by a decrease in fossil spores of *Sporormiella*, an obligatory coprophilous fungus occurring exclusively on dung from herbivorous animals, suggesting a decrease in megafauna probably due to hunting [12]. The first permanent settlements in the highlands are indicated by charcoal from increased burning and ceramics, dated to the thirteenth century AD [1,13]. Livestock proliferation on Madagascar is first indicated by a rise in *Sporormiella* spores *ca* 1100 YBP [12]. The earliest remains of domestic dog includes bones excavated in Rezoky in western Madagascar, dating to 500–700 YBP [1].

Ancient long-distance contacts across the Indian Ocean are demonstrated by genetic, linguistic and cultural evidence, connecting East Africa with Southeast Asia, the Indian subcontinent and Southwest Asia. However, research is still limited and few details are known about these contacts, particularly before the establishment of the trading societies of the Swahili coast [14]. Early contacts between East Africa and Indonesia are suggested by archaeobotanical and archaeozoological evidence [15]. There is, for example, ancient presence in Africa of Indonesian food plants such as banana (at least 5000 YBP) [16–18], and of water yam and taro [15,19]. At the time when Madagascar was first populated there was extensive trade across the Indian Ocean, which has later left traces in Madagascan culture also from, e.g. Southwest Asia and India [20–22].

Genetic studies based on mitochondrial DNA (mtDNA) and Y-chromosome DNA clearly reveal that Madagascar has an admixed human population of African and Indonesian origins, with approximately equal contribution of both maternal and paternal lineages from each source [23,24]. The Asian ancestry is more conserved among the inland than the coastal populations [23,24], which is consistent with a clear phenotypic difference between highland ‘Indonesian’ and lowland ‘African’ peoples [8]. In agreement with the linguistic evidence for the origin of the Malagasy language, Hurles *et al.* [23] identified Banjarmasin in southeastern Borneo as the most likely Asian source of the paternal ancestors of the Malagasy people.

At the time when Austronesian people arrived in Madagascar (i.e. 1500–2000 YBP), dogs had been present in East Asia for several millennia [25,26]. Also by this time, the domestic dog had recently arrived in sub-Saharan Africa [27,28]. Madagascan dogs could, therefore, have originated from either of these regions. We have previously shown that dog populations related to Austronesian speaking peoples in Indonesia and ancient Polynesia carry a signature haplotype (Arc2) at high proportions [29]. This haplotype was found in 41% of dogs in Kalimantan and Bali and in 68% of ancient samples from across Polynesia, but was absent outside Southeast Asia and Oceania [25]. Thus, there is a considerable difference between the gene pools of dogs in Indonesia/Polynesia and Africa, which presents us with the opportunity to identify the proportion of genetic ancestry of the Madagascan dogs from either of these two populations.

Here, we perform, to our knowledge, the first study of mtDNA in Madagascan dogs, comparing a large sample from across Madagascar with potential ancestral populations in Island Southeast Asia and sub-Saharan Africa. We investigate the proportion of ancestry from Asian dogs brought with the Austronesian expansion and from African dogs in subsequent contacts, illuminating Madagascan cultural origins as well as the initial dispersal of dogs across the world.

## 3. Material and methods

### 3.1 Samples

Three hundred and twenty nine samples of dogs from Africa (145 samples from Madagascar and 184 samples from the African mainland) were sequenced for a 582 bp fragment of the control region of the mitochondrial genome (positions 15 458 to 16 039) and compared with 2470 dogs from across the world published in earlier studies [25,30–34]. Dogs were sampled primarily in rural areas with low influx of foreign dogs, and avoiding known relatedness among individuals. The geographical origin of dogs for regions specifically studied were as follows (see also figure 1, table 1 and the electronic supplementary material, table S1): Africa—Madagascar coastal: northern (*n*=25), dry southern (*n*=11), Southeast (*n*=10), Southwest (*n*=8); Madagascar highland (*n*=38), misc. (*n*=54); East sub-Saharan Africa: Botswana (*n*=8), Kenya (*n*=9), Lesotho (*n*=6), Mozambique (*n*=8), South Africa (*n*=119), Tanzania (*n*=43), Uganda (*n*=148), Zambia (*n*=11); West sub-Saharan Africa: Benin (*n*=3), D. R. Congo (*n*=4), Gambia (*n*=4), Namibia (*n*=119), Sudan (*n*=3), misc. (*n*=8); North Africa: Algeria (*n*=1), Egypt (*n*=43), Mali (*n*=1), Morocco (*n*=19), Tunisia (*n*=9), misc. (*n*=12); Indonesia—Bali (*n*=61), Kalimantan (*n*=65), Sulawesi (*n*=3), misc. (*n*=2); Polynesia (archaeological specimens analysed for a shorter region (263 bp; positions 15 458–15 720))—Cook Islands (*n*=2), Hawaii (*n*=4), New Zealand (*n*=13); India (*n*=59); Taiwan (*n*=52); the Philippines (*n*=36); Australia (Dingo)—Victoria (*n*=35), New South Wales (*n*=110), Northern Territory (*n*=3), Queensland (*n*=44), South Australia (*n*=6), Western Australia (*n*=29), misc. (*n*=5); Southwest Asia—Afghanistan (*n*=6), U.A.E. (*n*=1), Iran (*n*=150), Israel (*n*=25), Kazakhstan (*n*=2), Kyrgyzstan (*n*=2), Saudi Arabia (*n*=5), Syria (*n*=7), Tajikistan (*n*=1), Turkey (*n*=111), Uzbekistan (*n*=1), misc. (*n*=24); Europe—Britain (*n*=121), North continent (*n*=142), South continent (*n*=117); Scandinavia (*n*=64), misc. (*n*=6).

**Table 1.** Genetic diversity in Madagascar and other most relevant populations. (*n*, number of samples; UT/UTd, proportion of individuals carrying a universal haplotype (UT) and a universal haplotype-derived (UTd); shared, proportion of individuals carrying a haplotype shared with Madagascan dogs; shared/derived, proportion of individuals carrying a haplotype shared with Madagascan dogs or a haplotype probably derived from a shared haplotype; non-UT shared, proportion of individuals carrying a non-universal haplotype that share haplotype with Madagascan dogs.)

population	*n*	UT	UTd	shared	shared/derived	non-UT shared
Madagascar total	145	79.3	93.8	—	—	—
Madagascar highlands	38	78.9	97.4	—	—	—
Madagascar coast	60	70.0	91.7	—	—	—
Indonesia	131	22.1	26.7	25.5	64.1	0
Polynesia	19	—	—	1.4	38.6	0
Southeast Borneo	65	29.2	36.9	22.1	55.9	0
sub-Sahara	490	78.8	92.2	89.7	100	60.0
East sub-Sahara	352	80.4	94.3	88.3	100	53.3
upper East sub-Sahara	211	82.9	98.6	86.2	97.9	43.3
lower East sub-Sahara	141	76.6	87.9	69.0	98.6	50.0
West sub-Sahara	138	74.6	87.0	66.9	97.9	50.0
Southwest Asia	345	77.7	94.5	80.0	93.1	3.3
India	59	57.6	81.4	56.6	89.0	6.7
Europe	450	76.9	92.2	83.4	95.9	20.0

### 3.2 DNA extraction, amplification and sequencing

Samples were collected as hair samples (*n*=67) and buccal epithelial cell samples (*n*=262) on FTA^®^ cards (Whatman Inc.). Hair samples were extracted as described in Angleby & Savolainen [35] and buccal epithelial cell samples were extracted according to the manufacturer's instructions. PCR amplification, DNA sequencing and sequence analysis were performed as in Angleby & Savolainen [35].

### 3.3 Analysis of the sequence data

DNA sequences were edited using sequencing analysis (Applied Biosystems, Foster City, CA, USA). Assembly into contigs and further editing was performed using Sequencher 4.1 (Gene Codes Corporation, Ann Arbor, MI, USA). The novel haplotypes found in this study were deposited in GenBank under accession nos. KR069086 (haplotype A250) and KP295480–KP295494 (haplotypes A178, A245, A246, A248, A249, A251, A254, A258, A261, A262, A273, A274, B052, B055, and B056, respectively).

Phylogenetic relations between haplotypes were displayed in minimum-spanning networks drawn by hand based on genetic distances calculated with Arlequin v. 3.11 software [36]. The networks were based on mtDNA sequences from dogs in the specifically studied geographical regions, as well as from dogs sampled across the world [25,30–34], to display the global phylogeny of dogs.

The mutation rate for the 582 bp region was obtained from [25] based on the average genetic distance between dog/wolf and coyote in a phylogenetic tree, calibrated with the time for the separation between the wolf and coyote lineages. The exact calibration point for the wolf/coyote separation is not known, but it may have occurred 1.5–4.5 million YBP. This gives a rate of 1.1×10^−8^–4.3×10^−8^ substitutions per site per year, or 1 substitution per 40 000–155 000 years.

The time of arrival of dogs to Madagascar was estimated using the statistic *ρ* (the mean number of substitutions for a set of sequences to their common ancestral haplotype) [37], based on the mean distance of Madagascan sequences to the founder haplotypes, and the substitution rate. Because the separation time between wolf and coyote can only be given as a range of possible dates, the time estimate is also obtained as a relatively broad range of possible time.

Genetic distances among geographical regions were estimated as *F*_ST_ values based on nucleotide differences including indels using Arlequin v. 3.5.1.2 [36] software. The matrix of pairwise *F*_ST_ values was summarized in two dimensions using multidimensional scaling (MDS) analysis implemented in Statistica v. 11 software package (StatSoft, Tulsa, OK, USA).

## 4. Results

We analysed 582 bp of the mtDNA control region in 145 dogs from across Madagascar in order to investigate their origin (figure 1 and table 1). The Madagascan dog sample was compared with dogs from the two broad source regions for the human Malagasy population, Austronesian speaking populations in Indonesia and African populations in sub-Saharan Africa, and also with ancient samples from across Polynesia linked to the Austronesian expansion. These comparisons were performed in the context of a comprehensive sample of dogs from the Old World, including Europe (to detect the impact of, e.g. the colonial era), and India and Southwest Asia, which have also influenced the Malagasy culture.

**Figure 2.**
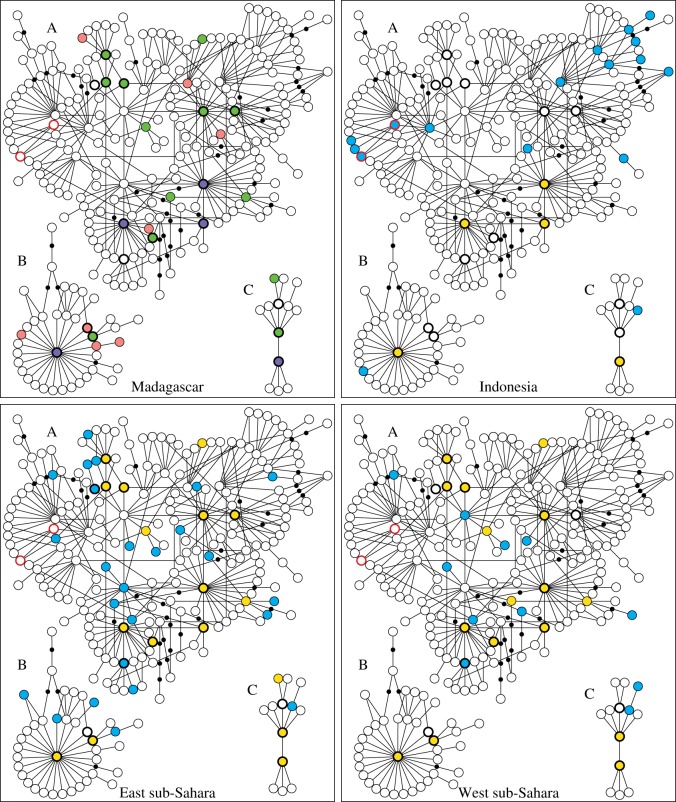
Minimum-spanning networks showing relationships between the haplotypes in the major mtDNA haplogroups A, B and C, and representation of the haplotypes in Madagascar, Indonesia, and East and West sub-Saharan Africa. Circles represent mtDNA haplotypes, lines connecting haplotypes represent a single substitution step, and black dots represent hypothetical haplotypes. The 17 universal haplotypes (UTs) are indicated with black bold outlining. Circles with red bold outlining represent haplotypes corresponding to the ‘Austronesian signature’ 263 bp haplotype Arc2. For Madagascar, pink circles represent haplotypes unique to Madagascar compared with the other three regions, purple represents haplotypes shared with both sub-Saharan Africa and Indonesia, and green represents haplotypes shared only with sub-Saharan Africa. Importantly, no haplotypes were shared exclusively between Indonesia and Madagascar. For Indonesia and East/West sub-Saharan Africa, yellow circles indicate haplotypes shared with Madagascar, and blue circles haplotypes not shared with Madagascar. For clarity of the picture, nine haplotypes are not shown (see the electronic supplementary material, figure S1).

The sub-Saharan African and Indonesian/Polynesian samples were considerably different, making it possible to investigate the extent of contribution from each of these populations to the Madagascan dog gene pool (figure 2 and table 1). The sub-Saharan African sample was similar to most populations across the Old World with 78.8% universally occurring haplotypes (UTs: haplotypes found in Europe, Southwest Asia as well as East Asia, and in most other regions of the Old World) and 92.2% UTds (‘UTs and UT-derived haplotypes’: UTs and haplotypes differing by a single substitution from a UT, probably deriving from the UT). By contrast, the Indonesian/Polynesian sample was quite divergent, with low proportions of individuals in Indonesia carrying a UT (22.1%) or UTd (26.7%), and 40% of the individuals in Indonesia and 68% of ancient Polynesian samples carrying the ‘Austronesian signature’ haplotype Arc2 (table 1; electronic supplementary material, table S1).

**Figure 3.**
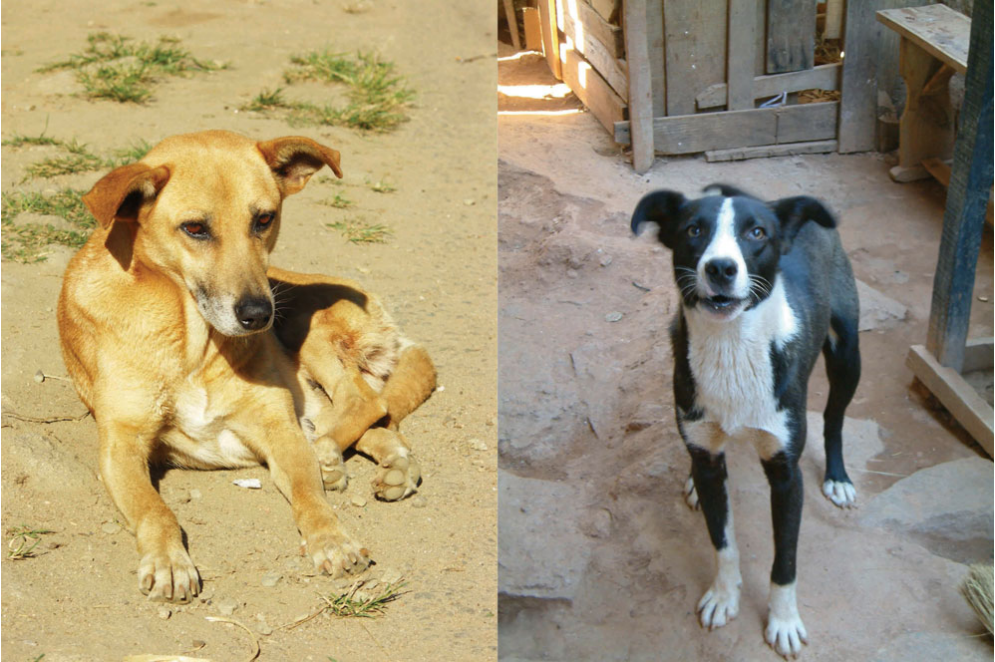
Indigenous dogs from Ranomafana (left) and Antananarivo (right), central east Madagascar. Photos by Lars-Göran Dahlgren.

In the Madagascan dog sample (figure 3), 26 haplotypes were detected, four of which unique to the island and carried by 4.1% of the individuals (figure 2 and table 2). In close agreement with sub-Saharan African samples, we found 79.3% UTs and 93.8% UTds (table 1). Madagascan dogs shared 18 of their 26 haplotypes (carried by 89.7% of the individuals) with dogs in sub-Saharan Africa (figure 2, tables 1 and 2). Four of the haplotypes (carried by 9.7% of the Madagascan dogs and by 46.7% of those carrying non-UTs) were exclusively shared between sub-Saharan Africa and Madagascar. All remaining haplotypes differed by a single mutation from the 18 shared haplotypes or (in one case) by one mutation from a haplotype unique to Madagascar. Thus, all 26 haplotypes found among Madagascan dogs were identical to, or possibly derived from, haplotypes found in sub-Saharan Africa, and may potentially have originated there.

**Table 2.** Haplotypes found in Madagascar, and their representation in other regions. (UT, universal haplotype; afr, exclusively shared between Madagascar and sub-Saharan Africa; u, unique in Madagascar; S, haplotype shared between Madagascar and the indicated region; d, haplotype differing by a single mutation from a shared haplotype and therefore possibly derived from the shared haplotype.)

HTs in Madagascar (*n*)	Indonesia (*n*=131)	Polynesia (*n*=19)	Southeast Borneo (*n*=65)	sub-Sahara (*n*=490)	East sub-Sahara (*n*=352)	West sub-Sahara (*n*=138)
A3 (1) UT	d	S	d	S	S	S
A5 (1) UT	—	S	—	S	S	S
A11 (15) UT	S	d	S	S	S	S
A16 (1) UT	—	—	—	S	S	S
A17 (35) UT	—	d	—	S	S	S
A18 (4) UT	S	—	—	S	S	S
A20 (1) UT	S	—	d	S	S	S
A22 (1) UT	—	d	—	S	S	d
A24 (1)	—	d	—	d	d	d
A27 (7) UT	d	—	—	S	S	S
A32 (8) afr	d	—	d	S	S	S
A33 (2)	—	d	—	d	d	d
A71 (4)	—	—	—	S	S	S
A252 (2) afr	—	—	—	S	d	S
A253 (1) afr	—	—	—	S	S	S
A273 (1) u	d	—	—	d	d	d
A274 (1) u	—	—	—	d	d	d
B1 (13) UT	S	—	S	S	S	S
B6 (10) UT	d	—	d	S	S	d
B27 (3) UT	d	—	d	d	d	d
B36 (3)	d	—	d	d	d	d
B55 (2) u	d	—	d	d	d	d
B56 (2) u	d	—	d	d	d	d
C1 (19) UT	d	—	d	S	S	d
C3 (4) UT	S	—	S	S	S	S
C16 (3) afr	—	—	—	S	S	—

By contrast, only five of the haplotypes found in Madagascar (all of which UTs), carried by 25.5% of Madagascan dogs, were shared with Indonesia, and totally 64.1% of the Madagascan dogs carried a haplotype which was shared or possibly derived (figure 2, tables 1 and 2). Compared with the ancient Polynesian samples, only two haplotypes (both UTs, carried by 1.4% of Madagascan dogs) were shared between Madagascar and Polynesia. It is especially notable that if UTs are ignored, not a single Madagascan individual had a haplotype shared with dogs from Indonesia or Polynesia, while 60.0% shared haplotypes with sub-Saharan Africa (table 1).

Furthermore, haplotype Arc2 dominates the mtDNA gene pools of the Austronesian dog populations of Indonesia and ancient Polynesia. It was found in 40% of dogs in Kalimantan and Bali and 68% of ancient samples from across Polynesia (electronic supplementary material, table S1). However, Arc2 was absent among Madagascan dogs (figure 2).

It is also worth pointing out that the Indonesian sample included 65 samples collected from within a 200 km radius from Banjarmasin in Southeast Borneo (figure 1). This area has been pinpointed the origin for the Austronesian language spoken in Madagascar, and its human population shown to carry an mtDNA pool which may be ancestral to that of the first Malagasy colonizers. The dogs from this region carried 10 haplotypes and only three of these (all of which UTs), carried by 22.1% of Madagascan dogs, were shared with Madagascar (tables 1 and 2).

Thus, except for universally occurring haplotypes, there was no sharing of haplotypes between Madagascan dogs and Indonesian or Polynesian dogs, and the ‘Austronesian signature’ haplotype Arc2 was entirely missing in Madagascar. Thus, no evidence was found supporting that the domestic dog would have been introduced to Madagascar in connection with the expansion of Austronesian speaking peoples from Island Southeast Asia. By contrast, 100% of the Madagascan dog samples had mtDNA haplotypes which may originate from Africa. An exclusively African origin is thus possible and, considering that the ‘Austronesian signature’ haplotype was completely absent in Madagascar, there is no indication even of a small contribution from Austronesian populations in Indonesia. Therefore, in contrast to the dual origin of the human population, Madagascan dogs seem to originate entirely from Africa.

Because of the high frequency of UTs in most parts of the world, in the western part of the Old World generally 75–80%, and because in Madagascar itself 79.3% of the dogs carried a UT, Madagascar has a high degree of haplotype sharing also with regions other than Africa. However, when UTs are ignored the sharing is much lower than for sub-Saharan Africa. Compared with India, 56.6% of the Madagascan dogs carried a shared haplotype and compared to Southwest Asia there were 80.0% Madagascan dogs sharing a haplotype (table 1; electronic supplementary material, table S1). However, ignoring the universally occurring UTs, only 6.7% of Madagascan dogs shared a haplotype with Indian dogs and 3.3% shared haplotypes with Southwest Asian dogs, and no haplotypes in Madagascar were shared exclusively with these regions. Therefore, an influence from India or Southwest Asia is possible, but there is no clear indication for this. A slightly higher degree of sharing is seen for Europe, with 83.4% of the Madagascan dogs carrying a haplotype shared with Europe, and 20.0% if UTs are ignored. One haplotype (B36) was exclusively shared between Europe and Madagascar, and haplotype A24 was shared only between Europe, Southwest Asia and Madagascar (electronic supplementary material, table S1). B36 has been found among Portuguese dogs [38], and A24 among dogs from Britain and France, all of which are countries with close historical contacts with Africa and/or Madagascar. Thus, the slightly higher degree of haplotype sharing with Europe possibly reflects historical contacts between Madagascar and Europe in the colonial era.

The direct source population(s) for the Madagascan dogs may be assumed to have been located somewhere along the African east coast, but the available sample material for this study included few samples from the actual coastal region. Possibly as a consequence of this, we did not find any region in sub-Saharan Africa to be especially genetically similar to Madagascar, but instead found comparable similarity to Madagascan dogs for several dog populations across sub-Saharan Africa. For example, compared with West sub-Saharan Africa (figure 1), 67% of Madagascan dogs carried a shared haplotype, but ignoring UTs 50% shared a haplotype, while compared with upper East sub-Saharan Africa, 86% of Madagascan dogs shared a haplotype, and ignoring UTs 43% shared a haplotype (table 1). Out of the four haplotypes that Madagascar shared uniquely with Africa, three were shared with West sub-Saharan Africa and two with upper East sub-Saharan Africa. Neither did analysis of F_ST_ give any clear indication of Madagascan dog ancestry (electronic supplementary material, figure S2). Except for Indonesia, and to some extent Madagascar, the populations from across the Old World were similar, with very little correlation between genetic and geographical distance. This probably reflects the universal sharing of haplotypes (the UTs): 75–80% of the individuals in each population share their haplotypes with dogs in practically all other populations, implying that random variation in frequencies of the UTs dominates these analyses. In MDS, Madagascar falls closest to upper East sub-Saharan Africa and Britain in dimension 1, followed by West sub-Saharan Africa (electronic supplementary material, figure S2*b*). However, with a stress value of more than 0.15 for this map, a relatively high level of distortion in the input genetic distances is indicated.

Within Madagascar there were relatively small genetic differences among regions; 81.6% of the individuals in highland Madagascar and 78.3% in the coast carried a haplotype shared between the two regions. The four haplotypes that were unique to Madagascar had restricted distribution: A273 and A274 were found in the north coast, B55 in the south coast and B56 in the highlands.

The sharing of 18 haplotypes between Madagascar and Africa indicates a minimum of 18 female lineages in the founding population(s), considerably more than in other island dog populations with a possible relation to the Austronesian expansion; Australian dingo founders probably carried a single mtDNA founder haplotype and ancient Polynesian dogs only two [31]. The time of introduction of dogs to Madagascar can be roughly estimated by calculating rho (*ρ*), representing the mean distance of the Madagascan sequences to the founder haplotypes [37]. We assume the haplotypes shared with Africa to be founder haplotypes, and the four unique haplotypes to be derived haplotypes, which gives *ρ*=0.055. Assuming a mutation rate of 1 substitution per 40 000–155 000 years as previously estimated [25], this suggests that dogs would have arrived in Madagascar 2200–8500 YBP, that is earlier than indicated by the archaeological evidence. However, the calculation of *ρ* is sensitive to failure of identifying all founder haplotypes. If any of the four haplotypes unique to Madagascar is present in Africa but was not included in the African sample, and thus is actually a founder haplotype brought from Africa rather than a derived haplotype, the calculated time of introduction is an overestimation.

## 5. Discussion

This study provides, to our knowledge, the first clear indications of the routes of introduction of dogs to Madagascar, filling in the emerging picture of the dispersal of dogs across the globe, and of the origin of the earliest domestic animals in Madagascar. We show that the gene pools of dogs in Indonesia/Polynesia and Madagascar are largely non-overlapping, and that the Madagascan sample lacks the ‘Austronesian signature’ haplotype carried by 40% of Indonesian and 68% of Polynesian dogs. By contrast, all the investigated Madagascan haplotypes could be linked to haplotypes found in sub-Saharan Africa.

A small component of the Madagascan diversity seems to have originated from Europe, probably in the colonial era. Other genetic contribution from Southwest Asia or India, as may be anticipated considering the long history of sea trade between the African east coast and the Persian Gulf region, and later the Indian subcontinent [21,22], is not indicated but cannot be ruled out. Therefore, our data gives a distinct indication that the domestic dog population in Madagascar was founded solely from African dogs with no contribution from Austronesian related populations. It also suggests some European influence, and while genetic contribution from Southwest Asia and India is not indicated, it remains possible.

These results are in general agreement with other datasets. Linguistic evidence suggests an Indonesian origin for the Malagasy language [5–7] and human population genetics suggests a dual Indonesian and African origin for the Malagasy people [23,24], but archaeological evidence suggests an African origin for the material culture [3]. An African origin of Madagascan dogs, as part of the material culture, agrees with this scenario. The African origin of domestic animals is also supported by linguistic evidence showing that the word for dog, as well as most other animals and domesticates in Madagascar, is adopted from African Bantu languages [2,8,9].

Dogs, together with pigs and chickens, were important domestic animals in the Austronesian culture [4]. Therefore, it would be expected that dogs were brought in in the colonization of major new areas, and a seemingly total absence in Madagascar of dogs with Austronesian heritage is therefore surprising. One possible explanation could be that the migration from Indonesia was a very limited event bringing a small number of colonizers to Madagascar in just a few journeys. However, this is contradicted by the relatively high diversity in the human Malagasy maternal and paternal lineages with Indonesian origin [23,24] that indicates introduction of large numbers of people, possibly in several successive waves rather than a severe genetic bottleneck in the founder population. However, regardless of the number of voyages from Indonesia, it is possible that if dogs were brought along in these long journeys, they died from the hardship or were used as a food source. There is also a possibility that Austronesian haplotypes carried by a limited number of Indonesian dogs were swamped by massive influx of dogs from Africa; a scenario potentially to be tested by analysis of ancient DNA from the earliest archaeological dog remains.

Another possibility is that Austronesian people colonized Madagascar via initial settlements on the African east coast, and hereby introduced African rather than Indonesian dogs to Madagascar. At the time when Madagascar was first populated, extensive trade existed across the Indian Ocean [20]. Because of these transoceanic contacts it has been suggested that the colonization of Madagascar took place via East Africa, and possibly dogs were introduced to Madagascar this way. There are no Austronesian or Malagasy people currently living on the East African coast [4,39,40], but a possible explanation for this could be that the Bantu expansion swept away earlier populations after it reached the East African coast approximately 2000 YBP. It is not clear when dogs were introduced from Africa, whether it was at the first arrival of Austronesian speaking people or later with the subsequent African migrations to Madagascar. However, it is noteworthy that the genetic diversity observed in Madagascan dogs is considerably higher than for other island dog populations possibly related with the Austronesian expansion, e.g. the Australian dingo and ancient Polynesian dogs [31,41–43]. This might indicate that the introduction of dogs from Africa was a process extended over time.

This study adds one more piece to the puzzle of the origins of Madagascan culture, but the picture is still fragmented. More extensive sampling of coastal eastern Africa and analysis of additional genetic markers will be useful to pinpoint the African origins of the Madagascan dogs and reveal whether they have the same origin as the African ancestors of the human population or were picked up further north in putative colonies on the east coast. Hopefully, genetic studies of other domestic animals tightly linked to Austronesian culture, i.e. the pig and the chicken, will also be performed to reveal if these domesticates share an African ancestry with the dogs, or were instead brought from Indonesia. This would help to draw a broader picture of this exceptional cross-oceanic movement of people, and of the universal expansion of the farming culture.

## Supplementary Material

Figure S1. Minimum-spanning networks showing the relationships between the haplotypes in the major mtDNA haplogroups A, B and C.

## Supplementary Material

Figure S2. MDS plot of pairwise FST values calculated from dog mtDNA control-region sequences for different world regions.

## Supplementary Material

Table S1. Total sample list.

## Data accessibility

DNA sequences corresponding to the haplotypes introduced by this study are deposited in GenBank under accession nos. KR069086 and KP295480–KP295494. All supporting information is uploaded as the electronic supplementary material and may be reached in Data Supplement.
