# Supplementary figures and images for: African origin for Madagascan dogs revealed by mtDNA analysis
Source: R Soc Open Sci. 2015 May 20;2(5):140552. doi: 10.1098/rsos.140552 (PMC4453261; doi:10.1098/rsos.140552)

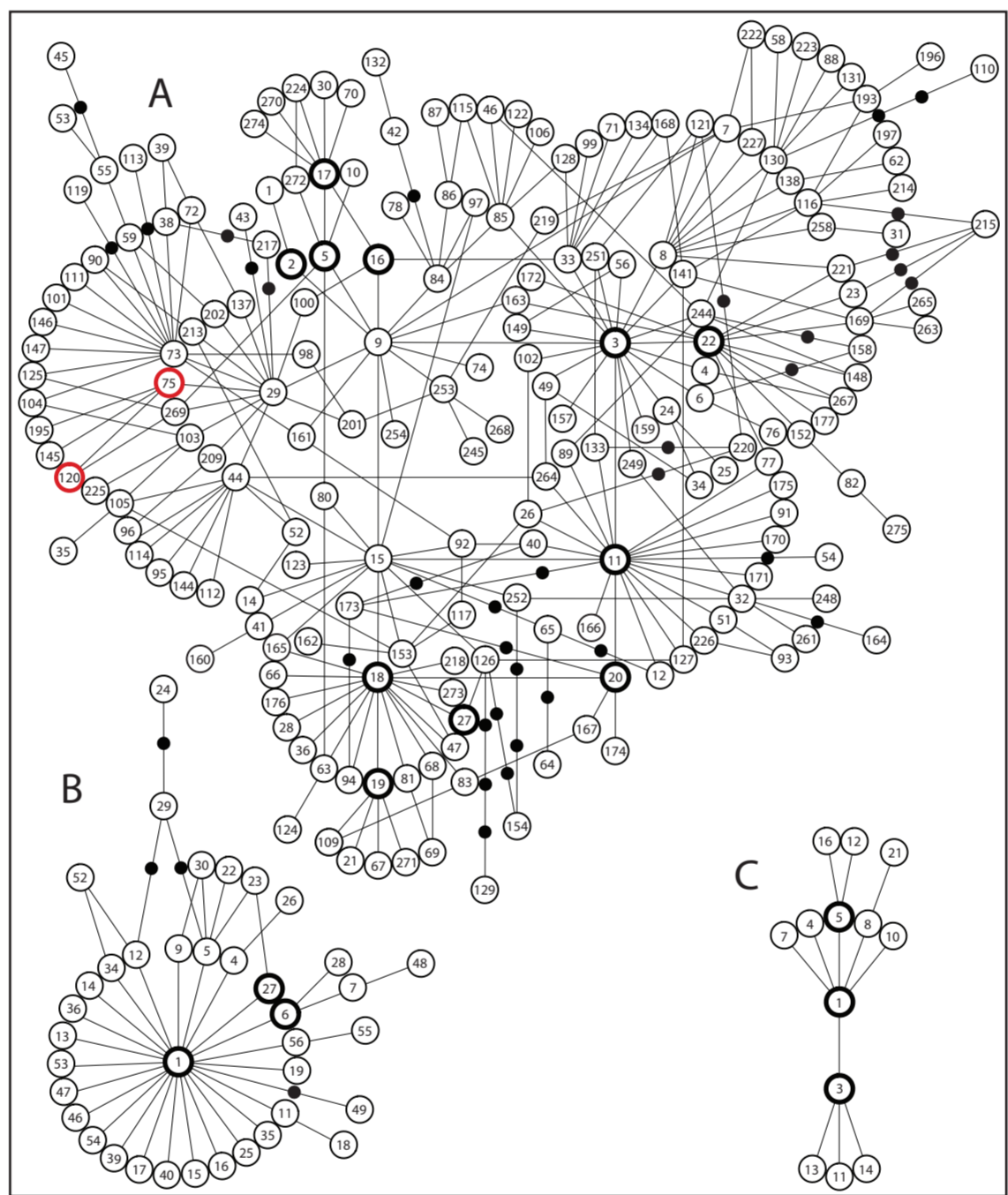

Supplement: Figure S1. Minimum-spanning networks showing the relationships between the haplotypes in the major mtDNA haplogroups A, B and C. [file rsos140552supp1.pdf]

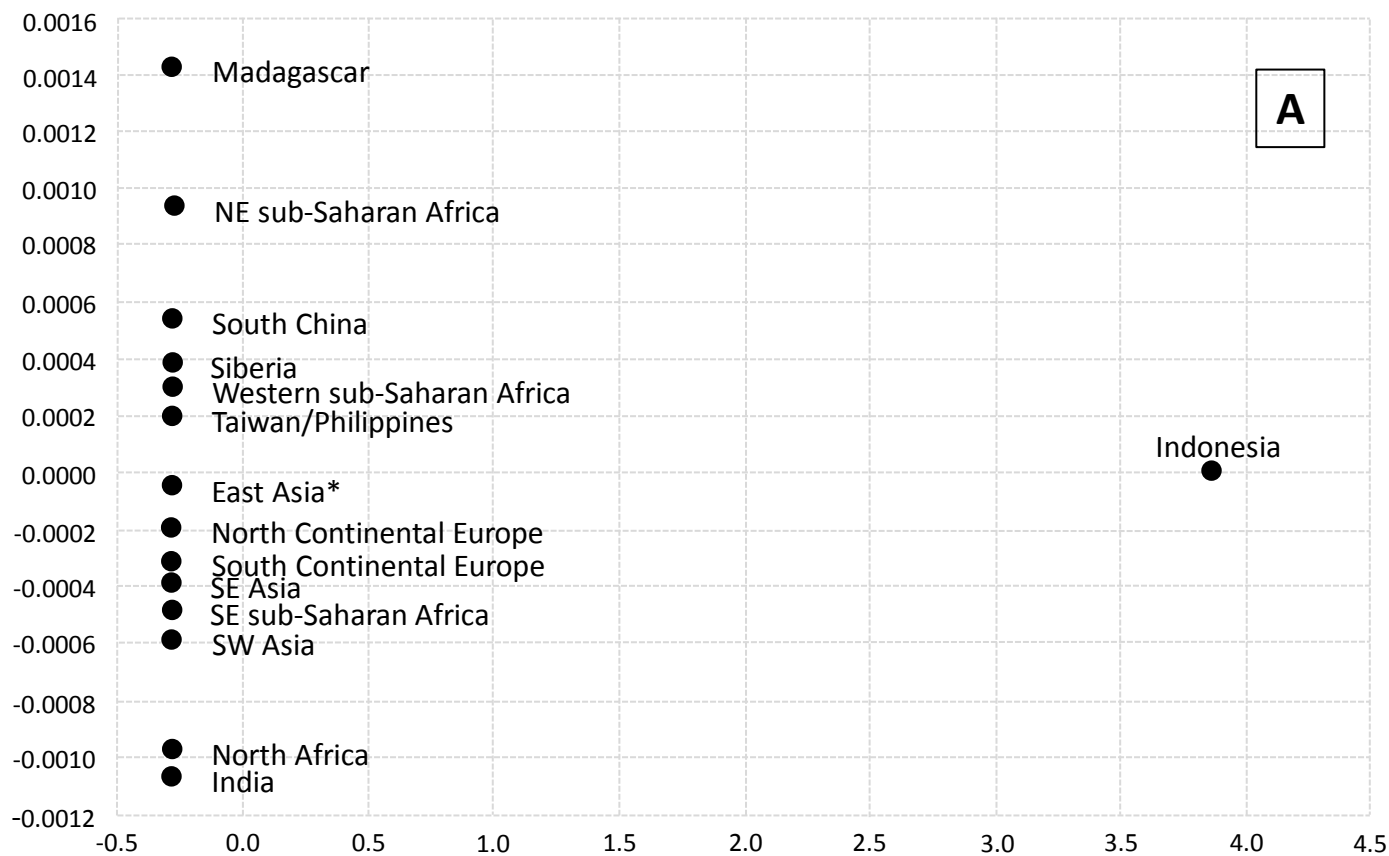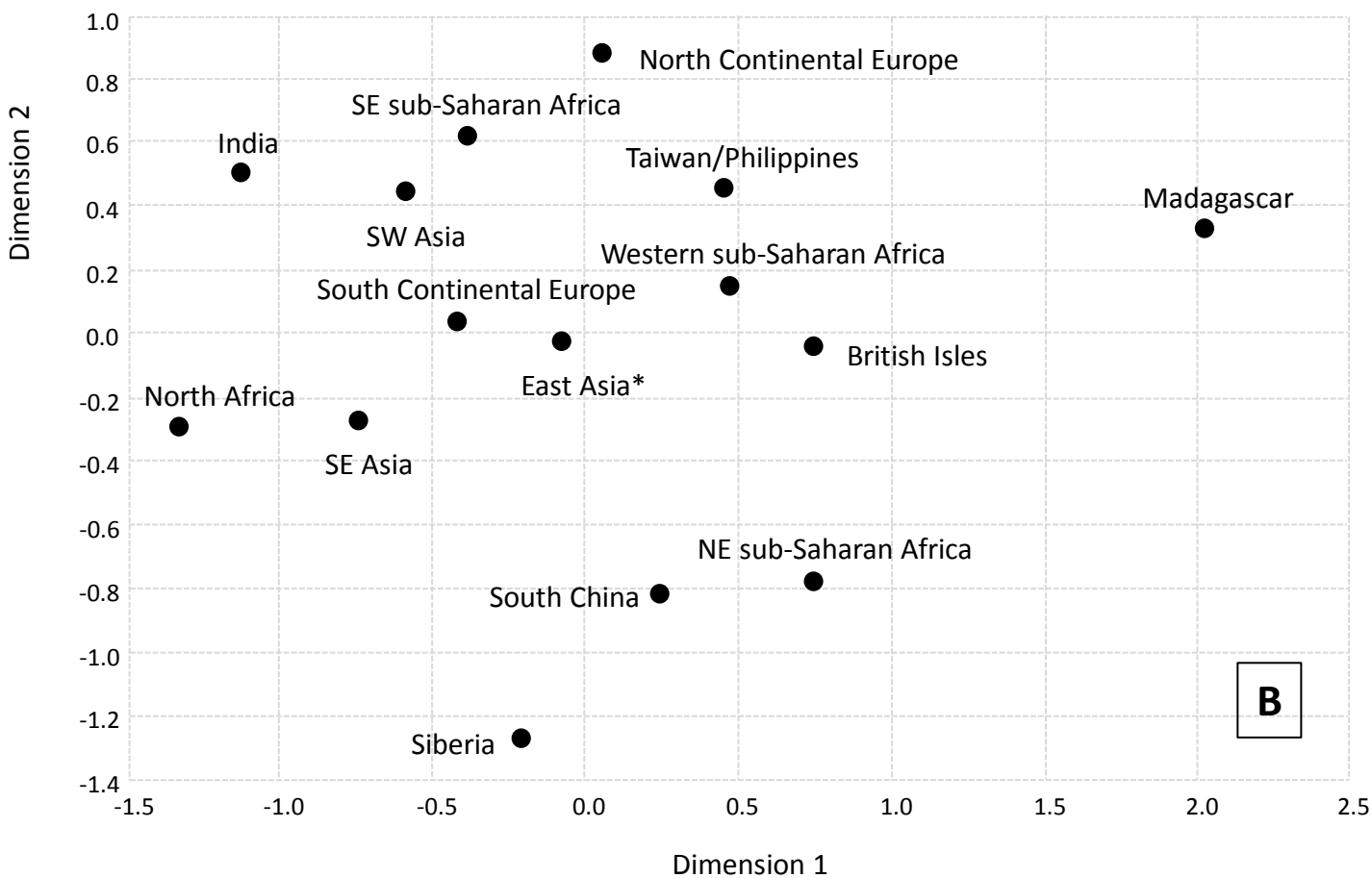

Supplement: Figure S2. MDS plot of pairwise FST values calculated from dog mtDNA control-region sequences for different world regions. [file rsos140552supp2.pdf]
